# Notes on Operative Dentistry and Its Advancement

**Published:** 1883-11

**Authors:** George H. Perine

**Affiliations:** New York


### NOTES ON OPERATIVE DENTISTRY AND ITS ADVANCEMENT.

BY GEORGE H. PERINE, NEW YORK.

Operative dentistry consists, so to speak, of particles, which when combined constitute an important and extensive branch of the science. Of late years the interest in the study of dental pathology has greatly increased, which certainly speaks well for the dental practitioner, for without pathological knowledge his efforts are at the best but random shots, the majority of which fall wide of the mark.

That the operation of filling teeth is one of very ancient origin is a fact which has been clearly demonstrated. For a time but little attention was paid by practitioners to the preservation of teeth which could not be filled without treatment. When the dentine was sensitive or the pulp exposed, they were either left to final decay or at once extracted. Through the efforts, however, of such men as Parmly, Wescott, and others, these erroneous ideas were eventually dispelled.

In 1846 Dr. Wm. H. Dwinelle presented a valuable paper on the preparation of teeth for filling, to the American Society of Dental Surgeons, and in 1850 he introduced transfer paper for articulating gold fillings, artificial crowns and artificial teeth, which is called articulating paper, and which for the purpose for which it is intended is invaluable.

In 1859, Dr. J. Taft published a valuable work, entitled "A Practical Treatise on Operative Dentistry," which was, and is still considered an able text book. At the time of its appearance it was the most elaborate treatise on the subject which had ever been printed. During the present year a work entitled "Notes on Operative Dentistry," from the pen of Dr. Marshall H. Webb, has been published, which is worthy of the most careful study.

At the present time, when the filling of teeth is more universally practiced than ever before, the treatment of such teeth is undertaken with a recognition of their pathological condition, and the operations are made to conform to the indications thus observed.

The treatment of deciduous teeth is also receiving its due share of attention. These were formerly regarded with much indifference, and little or no attention was bestowed upon them. It has been found, however, that their preservation is a matter of much importance, exerting great influence upon the permanent teeth, and they should be treated in a similar manner, except that for filling, plastic substances are employed.

It is a question who first introduced the practice of filling the roots of teeth. Dr. B. T. Longbotham recommended it as early as

1801, but it is believed by many that the credit is due to Dr. F. H. Badger, whom Dr. John S. Clarke, in 1843, declared to be the first to make nerve fillings, and the first also to introduce cylinder fillings. It is also claimed for Dr. James Taylor, that he was the originator of the latter method, but to Dr. Clarke, however, is due the credit at least of improving the method of making and introducing the cylinders.

At a meeting of the New York Dental Association in 1861, during a discussion upon the method of filling nerve cavities, a member stated that in preparing a root for the engrafting of an artificial crown, he discovered that the nerve cavity was perfectly filled and in good condition. Upon inquiry he was informed that the tooth had been filled by Dr. Hudson, nearly thirty years before, and that the nerve had been removed by a heated instrument. From this statement it would appear that the filling of the nerve cavity was an operation practiced when the specialty was exceedingly young in this country, and that some of the practitioners of half a century ago possessed in some branches of the profession a knowledge equal to that of the present day. The profound study and thoroughness which marked the efforts of some of the pioneers of the science, is worthy of general imitation, even in this advanced age.

Before speaking of the various materials employed for filling teeth, it will perhaps be as well for us to refer to some of the agents used in drying, and keeping moisture from the cavity previous to, and during the introduction of the filling.

Dr. A. Severance is said to have been the first to introduce prepared cotton for drying cavities. The hot air blow-pipe was suggested for this purpose by Dr. Geo. Watt, in 1855, who, with Dr. J. Taft, made the first hot air blow-pipe used. About this time Dr. A. M. Leslie suggested the adoption of asbestos bulbs of various sizes. Bibulous paper and spunk were also used, and many other plans and materials have been resorted to for this purpose by practitioners. Dr. John B. Rich claims to have used the gold plate coffer dam as early as 1836. In 1850 Dr. Dwinelle recommended the wax coffer dam, and Dr. A. Blakesley about the same period suggested the use of oil silk in conjunction with napkins. D. Geo. A. Mills favored the employment of a plaster coffer dam built up about the tooth, but to Dr. S. C. Barnum the specialty is more greatly indebted for the introduction, in 1864, of the rubber dam, which was at once adopted by practitioners generally. It has proved simple, as it is in its character one of the most valuable contributions to the advancement of operative dentistry.

The materials used for fillings are numerous. Gold, as in past ages, constitutes to-day the favorite essential. Gold foil for dental employment was, we believe, first manufactured in this country by Mr. M. Bull, in 1872. Chas. Abbey soon after joined him, and together they opened an establishment in Philadelphia. There are at this time several manufacturers of dentists' foil in the United States. Practitioners differ in their opinions regarding the most desirable properties of the gold employed. Some prefer cohesive, while non-adhesive is held in high esteem by others ; and the same difference of opinion as to weight exists, some preferring heavy and others light foils, made into rope, ribbon, or pellet form. Cylinders and blocks are now furnished by the manufacturers ready for use.

(To be concluded in December Number.)

